# Bridging methodological gaps in avian cytogenetics: comprehensive and optimized protocols for chromosomal preparation in birds

**DOI:** 10.1007/s10577-026-09803-8

**Published:** 2026-05-18

**Authors:** Marcelo de Bello Cioffi, Gustavo Akira Toma, Princia Grejo Setti, Jhon Alex Dziechciarz Vidal, Guilherme Mota-Souza, Marie Altmanová, Edivaldo Herculano Correa de Oliveira, Rafael Kretschmer

**Affiliations:** 1 Departamento de Genética E Evolução, Universidade Federal de São Carlos, 13565-905 São Carlos, SP Brasil; 2 Institute of Animal Physiology and Genetics, Czech Academy of Sciences, 27721 Liběchov, Czech Republic; 3 Seção de Meio Ambiente, Instituto Evandro Chagas, Ananindeua, PA 67030-000 Brazil; 4 Instituto de Ciências Exatas E Naturais, Universidade Federal Do Pará, Belém, PA 66075-110 Brazil; 5 Laboratório de Citogenética E Evolução, Departamento de Genética, Instituto de Biociências, Universidade Federal Do Rio Gran, de Do Sul, Porto Alegre, RS 91509-900 Brazil

**Keywords:** Avian cytogenetics, Cell culture techniques, Mitotic metaphase chromosomes, Microchromosomes, Mitotic index enhancement

## Abstract

This methodological study outlines effective and standardized procedures for obtaining high-quality chromosomal preparations in birds, which display complex karyotypes with a large number of microchromosomes. We describe every stage from sample obtaining and culture setup to the successful acquisition of metaphase chromosomes appropriate for in-depth analysis by combining three main methodologies (fibroblast, lymphocyte, and bone marrow cultures) into a coherent and methodical workflow. Furthermore, we offer thorough troubleshooting instructions to tackle typical problems, such as low mitotic indices, poor chromosome spreading, contamination, and suboptimal chromosomal morphology. We also focused on repeatability and adaptation across several bird taxa, taking into consideration species-specific differences in their body sizes, cell development, and chromosomal behavior, extending beyond standard procedure improvement. The incorporation of diverse methodologies into a unified framework offers avian cytogenetics researchers a readily available and practical workflow. Ultimately, our approach enhances the integration of cytogenetic data with novel genomic resources, facilitates the re-evaluation of chromosomal counts, and enables more precise karyotype characterization. This integration is crucial for advancing our understanding of chromosomal evolution, avian genome architecture, and the broader mechanisms underlying avian speciation and diversification.

**Supplementary Information:**

The online version contains supplementary material available at 10.1007/s10577-026-09803-8.

## Introduction

Avian cytogenetics has developed through more than a century of methodological innovation, yet it remains one of the most technically challenging groups of vertebrate chromosome biology. Unlike most other amniotes, birds possess a unique chromosomal structure characterized by high diploid numbers (2n) and numerous small microchromosomes, making it essential to prepare their chromosomes with outstanding precision. The typical avian ancestral karyotype is characterized by a clear division between a small number of large macrochromosomes and a large set of microchromosomes. The karyotypic organization now widely recognized as the ancestral condition for birds comprises approximately 40 chromosome pairs (2n = 78–80), with ~ 10 pairs of macrochromosomes and ~ 30 microchromosomal pairs (smaller than 20 Mb) (reviewed in Kretschmer et al. 2018). The microchromosomal complement encompasses the smallest elements, often referred to as “dot chromosomes” in recent high-resolution assemblies (Huang et al. 2023).

Guyer (1902) was among the first to investigate bird cytogenetics, studying spermatogenesis in the domestic pigeon (*Columba livia*) and the Eurasian collared dove (*Streptopelia decaocto*). These pioneering efforts, like most avian cytogenetic studies of the early twentieth century (e.g., Loyez 1906), relied solely on histological sections of gonadal tissue. Although such approaches provided valuable insights into meiosis, they were fundamentally limited in their ability to resolve individual chromosomes, accurately determine diploid numbers, or characterize chromosome morphology. As a result, advancements in avian cytogenetics during this era were gradual and inconsistent, and early chromosome counts frequently contained inaccuracies or were incomplete. The domestic chicken (*Gallus gallus*) subsequently emerged as the main model for avian cytogenetic research and has played a pivotal role in shaping our understanding of bird genome organization. However, even in this intensively studied species, establishing the correct diploid number proved remarkably difficult. Early estimates ranged from as few as six chromosome pairs in 1906 (Loyez 1906) to 39 pairs by 1944 (Yamashina 1944), when the currently accepted diploid number of 2n = 78 was finally established. This long revision process underscores the profound impact of technical limitations, particularly the difficulty of visualizing numerous small chromosomes. Similarly, the ZW sex chromosome system in birds was not formally described until 1961 (Frederic 1961), further illustrating how technical limitations of early chromosome preparations constrained progress in avian cytogenetics.

Despite substantial methodological advances over subsequent decades, inconsistencies in reported diploid numbers have persisted across avian taxa. A striking example is the helmeted guineafowl (*Numida meleagris*), for which diploid numbers of 2n = 76 (Beçak et al. 1971), 2n = 56 (Ebied et al. 2005), and 2n = 78 (O’Connor et al. 2019) have all been reported in different studies. Such discrepancies largely reflect the inherent technical difficulty of accurately discriminating avian microchromosomes, which are prone to loss, overlap, or misidentification in suboptimal metaphase preparations. Consequently, numerous early or low-resolution karyotypic descriptions have required subsequent revision or still require comprehensive re-evaluation.

Significant advancements in chromosome preparation methodologies, particularly the introduction of hypotonic treatments and the use of colchicine for mitotic arrest, represented a significant shift in avian cytogenetics. These innovations greatly improved metaphase spreading and chromosome visibility, revealing the organization of avian karyotypes composed of a small number of macrochromosomes and a large set of microchromosomes. The development of lymphocyte culture methods (Moorhead et al. 1960), along with the use of feather pulp and bone marrow cultures (Sasaki et al. 1968), expanded the range of tissues available for cytogenetic studies. These methods also improved the consistency of results across different research labs.

In recent decades, fibroblast cultures derived from skin biopsies or growing feathers have become one of the most widely adopted approaches for avian chromosome preparation. These cultures consistently produce high-quality metaphase spreads and allow for non-lethal sampling, making them especially useful for studying wild, rare, and/or endangered bird species (Bülau et al. 2021; Kretschmer et al. 2018). Protocols for establishing fibroblast cultures from avian embryos have also been proposed as an alternative source of mitotically active cells (Barcellos et al. 2022). Despite these advancements, avian cytogenetics continues to present technical difficulties, and inconsistencies in reported diploid numbers are still evident in the existing literature. This historical context emphasizes the critical influence of methodology on the development of avian cytogenetic knowledge. It also highlights the necessity of refined, standardized protocols that consistently yield high-quality chromosome preparations, thus reducing miscounts, enhancing reproducibility, and facilitating accurate reevaluation of avian karyotypic diversity. Moreover, notwithstanding progress in genome sequencing, high-quality avian cytogenetics remains indispensable for comprehending the structural organization of bird genomes. Specifically, avian microchromosomes, which are gene-rich and evolutionarily conserved (Galkina et al. 2017; O'Connor et al. 2019) pose challenges for reliable assembly from sequence data alone due to their small size, thereby necessitating cytogenetic approaches to resolve their organization. Accurate karyotype analyses yield significant insights into karyotype evolution, encompassing rearrangements of macro- and microchromosomes across avian lineages. In addition, chromosomal analyses further enable the detection of hybrid incompatibilities and reproductive barriers, thereby contributing to our understanding of speciation processes.

In this study, we aimed to compile, refine, and integrate the most optimized and comprehensive protocols currently available for avian mitotic chromosomal preparation, summarizing three principal approaches based on fibroblast, bone marrow, and lymphocyte cultures. These updated protocols are intended to facilitate reliable karyotypic analyses and support the re-evaluation of previously reported avian chromosome numbers. These protocols are widely used in our laboratories and have been consistently applied in cytogenetic studies conducted on different bird groups, demonstrating their robustness and reproducibility.

## Materials and reagents

A list of specialized items and instructions for preparing their working solutions is provided below:

- Lab equipment:
  - Analytical precision balance
  - Autoclave
  - Centrifuge (capable of ~ 800—1200 × g)
  - CO₂ incubator
  - Freezer (− 20 °C)
  - Hot plate (~ 50 °C)
  - Inverted microscope (for monitoring cell growth)
  - Laminar flow cabinet (biosafety cabinet) with UV light
  - Light microscope (with 100 × oil objective)
  - Pipette controller
  - Refrigerator (+ 4 °C)
  - Standard laboratory incubator (non-CO₂)
  - Ultrapure Water Purification System Milli-Q
  - Water bath (37 °C)
- Materials:
  -Adjustable micropipettes
  -Aluminum foil
  -Biohazard waste bags
  -Cell culture flasks (25 cm^2^) with vented polyethylene caps contain a 0.22 µm hydrophobic filter
  -Ethanol containers (for 70% and 100%)
  -Falcon tube racks (15 mL/50 mL)
  -Glass beakers (10 mL)
  -Glass microscope slides
  -Glass waste bottle with 10:1 water/sodium hypochlorite
  - Graduated glass flasks (100 mL)
  -Heparinized blood collection tube
  -Microcentrifuge tube racks
  -Scalpel blades
  -Scalpel handles
  -Scissors
  -Sterile 0.22 µm syringe filters
  -Sterile 1.5 mL microcentrifuge tubes
  -Sterile 15 mL Falcon tubes
  -Sterile 25 mL Glass Beaker
  -Sterile 50 mL Falcon tubes
  -Sterile 100 mL glass bottles
  -Sterile needles (e.g., 25–30G)
  -Sterile Petri dish (previously autoclaved)
  -Sterile pipette tips (various volumes)
  -Sterile plastic Pasteur pipettes
  -Sterile syringes (1 mL and 10 mL)
  -Volumetric flasks (1 L)
  -Tweezers/forceps
  −10 mL glass pipettes
- Reagents:
  -Acetic acid (glacial, ≥ 99.7%, Cat. No. 695092, Sigma-Aldrich, USA)
  -AmnioMAX-II complete medium (Cat. No. 11269016, Thermo Fisher Scientific, Waltham, MA, USA)
  -Concanavalin A from Canavalia ensiformis Type IV-S (Cat. No. C0412, Sigma-Aldrich, St. Louis, MO, USA)
  -Colchicine (Cat. No. C9754, Sigma-Aldrich, St. Louis, MO, USA)
  -Collagenase Type I (Cat. No. 17100017, Thermo Fisher Scientific, Waltham, MA, USA)
  -Dulbecco's Modified Eagle Medium (DMEM), high glucose with sodium bicarbonate, L-glutamine, and HEPES (Cat. No. 12430054, Thermo Fisher Scientific, Waltham, MA, USA)
  -Ethanol absolute (Cat. No. 1009831000, Merck, Darmstadt, Germany)
  -Ethanol 70% (Cat. No. EX0281, Merck, Darmstadt, Germany)
  -Fetal Bovine Serum (FBS) Qualified (Cat. No. 26140079, Thermo Fisher Scientific, Waltham, MA, USA)
  -Giemsa stain solution (Cat. No. 10092013, Thermo Fisher Scientific, Waltham, MA, USA)
  -Glycerol (C₃H₈O₃) (Cat. No.G7893, Merck, Darmstadt, Germany)
  -Hanks’ Balanced Salt Solution (Cat. No. H9394, Sigma-Aldrich, St. Louis, MO, USA)
  -Heparin sodium (Cat. No. H0200000, Merck, Darmstadt, Germany)
  -L-Glutamine (Cat. No. G7513, Sigma-Aldrich, St. Louis, MO, USA)
  -Lipopolysaccharide (Cat. No. L2880, Sigma-Aldrich, St. Louis, MO, USA)
  -Methanol (Cat. No. 322415, Sigma-Aldrich, USA)
  -Penicillin–Streptomycin-Amphotericin B (Cat. No. A5955, Sigma-Aldrich, St. Louis, MO, USA)
  -Phosphate-Buffered Saline 10 × (PBS) (Cat. No. 59331 C, Sigma-Aldrich, St. Louis, MO, USA)
  -Phytohemagglutinin M form (PHA-M) (Cat. No. 10576015, Thermo Fisher Scientific, Waltham, MA, USA)
  -Potassium chloride (KCl) (Cat. No. P9541, Sigma-Aldrich, St. Louis, MO, USA)
  -Potassium phosphate (KH₂PO₄) (Cat. No. 529568, Merck, Darmstadt, Germany)
  -RPMI-1640 Medium with L-Glutamine and HEPES (Cat. No. 22400089, Thermo Fisher Scientific, Waltham, MA, USA)
  -Sodium hypochlorite (Cat. No. 239305, Sigma-Aldrich, St. Louis, MO, USA)
  -Sodium phosphate (NaH₂PO₄) (Cat. No. 609773, Merck, Darmstadt, Germany)
  -Trypsin–EDTA (Cat. No. 25200072, Thermo Fisher Scientific, Waltham, MA, USA)
- Solutions to be prepared:
  **-Solution 1: DMEM supplemented with L-glutamine and antibiotics (DMEM Working solution)**

### Aliquot/storage container

Sterile 100 mL glass flask.

Prepare the DMEM working solution by supplementing DMEM medium with 1% (v/v) L-glutamine and 1.5% (v/v) penicillin–streptomycin-amphotericin B. For every 100 mL of DMEM medium, add 1 mL of L-glutamine (1%) and 1.5 mL of penicillin–streptomycin-amphotericin B solution (1.5%). Perform all procedures under sterile conditions in a laminar flow cabinet. Gently mix to ensure homogeneity and keep the flask closed, opening it only under sterile conditions. Store at 4 °C for up to two weeks and pre-warm to 37 °C before use.

**–Solution 2: 10 × Phosphate-Buffered Saline (PBS) or Hanks’ Balanced Salt Solution**

### Aliquot/storage container

Sterile 1000 mL glass flask.

**–Solution 3: Fetal Bovine Serum (FBS) aliquots**.

### Aliquot/storage container

Sterile 50 mL Falcon tube.

Aliquot sterile fetal bovine serum (FBS) into sterile Falcon tubes under aseptic conditions. Store at −20 °C and thaw at room temperature or in a 37 °C water bath before use. Avoid repeated freeze–thaw cycles.

–**Solution 4: 1 × Phosphate-Buffered Saline (PBS) or 1 × Hanks’ solution supplemented with antibiotics.**

### Aliquot/storage container

Sterile 50 mL glass flask.

Prepare 1 × PBS by diluting 10 mL of 10 × PBS in 90 mL of sterile Milli-Q water. Autoclave and allow it to cool. Under the laminar flow cabinet, add 1.5% (v/v) penicillin–streptomycin-amphotericin B and store the solution at 4 °C for up to four weeks.

–**Solution 5: Collagenase type I solution (4.5 mg/mL)**.

### Aliquot/storage container

Sterile 15 mL Falcon tubes (2 mL aliquots).

Dissolve 0.018 g of collagenase in 4 mL of DMEM culture medium to obtain a final concentration of 4.5 mg/mL. Filter-sterilize the solution through a 0.22 µm syringe filter under a laminar flow cabinet and supplement with 1.5% (v/v) penicillin–streptomycin-amphotericin B. Store aliquots of 2 mL into sterile 15 mL Falcon tubes and store at −20 °C.

–**Solution 6: Trypsin–EDTA solution.**

### Aliquot/storage container

Original sterile stock bottle or sterile 15 mL Falcon tubes.

Use sterile 0.25% trypsin–EDTA solution according to the manufacturer’s instructions. Store at −20 °C and thaw immediately before use.

–**Solution 7: Concanavalin A (ConA) Solution aliquot**.

### Aliquot/storage container

Sterile 1.5 mL microcentrifuge tube.

Dissolve 5 mg of Concanavalin A in 1 mL of sterile 1 × PBS. Filter-sterilize the solution through a 0.22 µm syringe filter under aseptic conditions. Store the aliquots at −20 °C.

–**Solution 8: Lipopolysaccharide (LPS) Solution Aliquot.**

### Aliquot/storage container

Sterile 15 mL centrifuge tubes (2 mL aliquots).

Dissolve 100 mg of LPS in 10 mL of Milli-Q water. Filter-sterilize the solution through a 0.22 µm membrane filter under aseptic conditions. Aliquot ~ 2 mL of the filtered solution into sterile 15 mL centrifuge tubes and store the aliquots at − 20 °C. Thaw the solution at room temperature before use and avoid repeated freeze–thaw cycles.

–**Solution 9: Supplemented RPMI (with concanavalin).**

### Aliquot/storage container

Sterile 15 ml centrifuge tubes.

Prepare the culture medium on the same day as sample collection. To obtain a final volume of 5 mL of supplemented RPMI supplemented medium, mix 3.785 mL RPMI with 1 mL fetal bovine serum (20%, v/v), 50 µL L-glutamine (1%, v/v), 50 µL penicillin–streptomycin-amphotericin B solution (1%, v/v), 50 µL lipopolysaccharide stock solution (10 mg/mL; final concentration 100 µg/mL), and 65 µL of ConA stock solution (5 mg/mL; final concentration 65 µg/mL). Store at 4 °C and pre-warm to 37 °C before use.

–**Solution 10: Supplemented RPMI (with Phytohemagglutinin).**

### Aliquot/storage container

Sterile 100 mL glass bottle.

Prepare the culture medium in a sterile glass bottle. For each sample, prepare a final culture volume of 5 mL by mixing 4.3 mL of RPMI medium with 500 µL of fetal bovine serum (10%, v/v), 50 µL of L-glutamine (1%, v/v), 50 µL of penicillin–streptomycin-amphotericin B solution (1%, v/v), and 100 µL of phytohemagglutinin (PHA-M; 2%, v/v). Store at 4 °C for up to two weeks and pre-warm to 37 °C before use.

–**Solution 11: Colchicine solution (0.01%).**

### Aliquot/storage container

Sterile 15 mL centrifuge tubes or autoclaved glass bottles.

Dissolve 0.05 g of colchicine in 500 mL of ultrapure Milli-Q water. Transfer the solution to an amber glass bottle or aliquot it into 15 mL Falcon tubes wrapped in aluminum foil to protect them from light. Store the aliquots at 4 °C for up to 1 year.

–**Solution 12: Hypotonic solution (0.075 M KCl).**

### Aliquot/storage container

Sterile 1000 mL glass bottle.

Dissolve 5.591 g of potassium chloride (KCl, Mw: 74.55 g/mol) in approximately 800 mL of distilled water. After complete dissolution, adjust the final volume to 1 L with distilled water in a volumetric flask. Store at 4 °C (for up to 4 weeks) and prewarm to 37 °C right before use.

–**Solution 13: Methanol–acetic acid (3:1) I fixative solutio**n

### Aliquot/storage container

Sterile 50 mL glass bottle.

Prepare fresh fixative by mixing methanol and glacial acetic acid in a 3:1 (v/v) ratio. Cool the fixative at −20 °C until it is ice-cold before use.

–**Solution 14: Giemsa (stock solution)**

### Aliquot/storage container

Sterile 100 mL glass bottle wrapped in aluminum foil.

Add 1 g of Giemsa to 54 mL of glycerol and heat to 60 °C. After cooling, add 84 mL of methanol and filter the solution. Aliquot it into 15 mL Falcon tubes wrapped in aluminum foil to protect them from light. Store the aliquots at room temperature (for up to 1 year).

–**Solution 15: Phosphate buffer (pH 6.8)**

### Aliquot/storage container

Sterile 100 mL glass bottle wrapped in aluminum foil.

- **Solution A:** Dissolve 4.0827 g of KH₂PO₄ in 500 mL of water.
- **Solution B:** Dissolve 3.1888 g of Na₂HPO₄ in 500 mL of water and mix well.

Prepare Solutions A and B separately. Then add Solution A gradually to Solution B until the pH reaches 6.8.

–**Solution 16: Giemsa working solution**

### Aliquot/storage container

Sterile 25 mL Glass Beaker.

Add 0.5 mL of Giemsa to 9.5 mL of phosphate buffer (Solution 15) and mix gently by pipetting. Prepare immediately before use.

## Sampling and culture establishment

The choice of culture method depends on the research objectives, available resources, and sample type. Fibroblast cultures generally produce metaphase spreads of higher quality and in greater numbers, making them particularly suitable for high-resolution karyotyping, such as describing new karyotypes or resolving ambiguous microchromosome counts. However, this method is the most time-consuming and costly one, requiring adequate laboratory infrastructure. Lymphocyte cultures from peripheral blood provide a faster and less resource-intensive alternative. They are ideal for screening multiple individuals, sex determination, or situations where only small sample volumes are available. Importantly, both blood and fibroblast sampling can be performed using minimally invasive procedures that do not require euthanasia of the animals. For specimens where sacrifice is already part of the study design, bone marrow cultures are particularly useful because they yield large numbers of rapidly dividing cells without the need for long-term culture. In certain cases, specimens may also originate from wildlife rehabilitation centers, where injured birds that cannot recover or be released back into the wild must be humanely euthanized according to established ethical guidelines. In these cases, the specimens can also be used for bone marrow cultures. Detailed sampling and methodological procedures for each of the approaches are provided below.

### Primary fibroblast cultures

#### Samples

Primary fibroblast cultures can be established from three main types of avian tissues: tissue biopsies, vascularized growing feather pulp, and whole embryos. These tissues provide reliable sources of fibroblasts and are commonly used for cytogenetic and cell culture applications.

i) **Tissue Biopsies**

Based on our previous experience with tissue processing and cell isolation, skin and lung tissues have consistently provided the most reliable results and are therefore recommended for this protocol.

The biopsies should be obtained using sterile surgical instruments, including scissors, tweezers (forceps), and scalpels. A small fragment of tissue (approximately 1 cm) should then be excised using a sterile scalpel blade and sterile forceps. For skin biopsies, the sampling surface must be disinfected with 70% ethanol before tissue collection. Immediately after collection, the tissue fragment should be transferred into a sterile 15 mL conical (Falcon) tube containing 5–10 mL DMEM working solution (Solution 1). Samples should be maintained at 4 °C and transported under refrigerated conditions until further processing.

ii) **Growing Feather (Feather Pulp)**

Actively growing feathers containing vascularized pulp should first be cleaned with 70% ethanol and, after drying, plucked from the sampled individual. The calamus region containing the pulp should be sectioned using sterile scissors and tweezers. The extracted feather pulp must then be transferred to a sterile 15 mL Falcon tube containing 5–10 mL of DMEM working solution (Solution 1) and maintained at 4 °C until processing. Juvenile birds typically possess numerous naturally growing feathers, which facilitate sampling. In adults, however, growing feathers may be less abundant; in such cases, 2–3 feathers can be plucked to induce regrowth, and newly growing feathers with vascularized pulp can be collected after approximately 15–20 days.

iii) **Embryo**

Primary fibroblast cultures are best established from embryos aged 8–14 days, as their small size facilitates handling and their tissues exhibit high mitotic activity. Fertilized eggs should be incubated at 36–38 °C until processing. Before embryo collection, disinfect the eggshell surface with 70% ethanol and transfer the egg to a laminar flow cabinet. Using sterile tweezers or scissors, carefully create a small opening in the shell while minimizing yolk leakage. Gently transfer the embryo to a sterile petri dish using tweezers. If necessary, rinse the embryo with sterile 1 × PBS or Hanks’ balanced salt solution (Solution 4). Process the embryo immediately. Alternatively, place tissue fragments in a sterile 15 mL Falcon tube containing 5–10 mL DMEM working solution (Solution 1) and store at 4 °C for up to 72 h before establishing the culture.

#### Establishing fibroblast cultures

Before beginning, ensure that all necessary materials (see the Materials and Reagents section) are available. Turn on the laminar flow cabinet and thoroughly wipe the work surface with 70% ethanol. Arrange the following items inside the cabinet: sterile Pasteur pipettes, sterile Petri dishes, sterile scissors, sterile tweezers, scalpel blades, cell culture flasks, and a waste bottle filled halfway with 1–10% sodium hypochlorite. Then turn on the UV light for at least 15 min to ensure sterility. Perform all subsequent steps under aseptic conditions, unless otherwise specified.

**Fig. 1:**
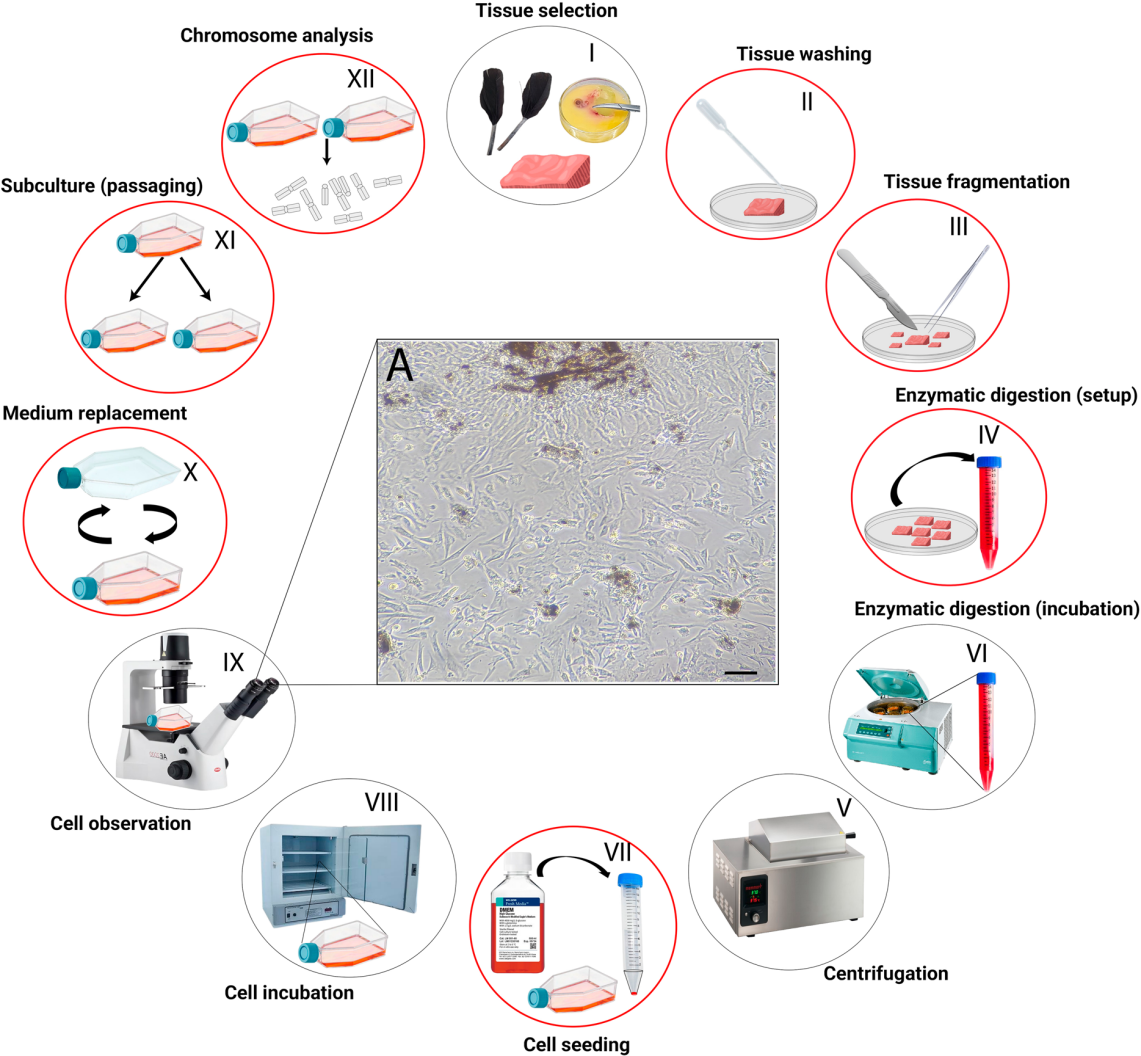
Workflow of fibroblast cell culture. Schematic overview of the fibroblast cell culture protocol from initial tissue selection (I) to chromosome analysis (XII). Red circles (II–IV, VII, X–XII) indicate steps requiring a strict aseptic environment, carried out in a laminar flow cabinet. II: Initial washing of tissues with 1% sodium hypochlorite followed by 1 × PBS. III: Mechanical fragmentation of selected tissues. IV–V: Transfer of tissue fragments to collagenase solution and enzymatic digestion during incubation. VI: Centrifugation of digested tissue to obtain cell pellets. VII: Transfer of resuspended cell pellets to culture flasks. VIII: Incubation at 37 °C in a humidified atmosphere containing 5% CO₂. IX: Visualization of adherent cells using an inverted phase-contrast microscope. X: Replacement of culture medium. XI: Subculturing fibroblast cultures. XII: Preparation and analysis of chromosomes from cultured cells. A: Representative images of cultured fibroblast cells (scale bar, 100 µm). The details of the chromosomal preparation are described in Fig. 4

For surface decontamination, dip all samples (except embryos) in 1% sodium hypochlorite for 1 min. Then wash the samples thoroughly at least three times (30 s each) in sterile 1 × PBS or Hanks’ balanced salt solution (Solution 4) to remove residual disinfectant. To perform the washes, place the biopsies in a clean Petri dish and add sufficient wash solution (Solution 4) using a Pasteur pipette to fully immerse the samples. Gently swirl the dish and carefully remove the solution, taking care not to aspirate the tissue (Fig. 1, step II). Next, cut the selected tissue into small fragments using sterile scissors and/or scalpel blades (Fig. 1, step II). Transfer the fragments into a sterile 15 mL Falcon tube containing 2 mL of collagenase solution (Solution 5) (Fig. 1, step IV). Remove the tube from the laminar flow cabinet and incubate it at 37 °C in a water bath or in an incubator for 3–8 h, depending on the tissue type (Fig. 1, step V) (See troubleshooting section). After digestion, add DMEM working solution (Solution 1) to a final volume of 10 mL and centrifuge for 8 min at 200 × g (Fig. 1, step VI). Return the tube to the laminar flow cabinet and carefully remove the supernatant without disturbing the cell pellet. Gently resuspend the cells in 4 mL of DMEM working solution (Solution 1) and 1 mL of FBS (Solution 3) to obtain a final volume of 5 mL, mixing slowly by pipetting (Fig. 1, step VII). Transfer the cell suspension to a sterile cell culture flask with vented polyethylene caps contain a 0.22 µm hydrophobic filter and incubate at 37ºC with 5% CO_2_ for 10–12 h (Fig. 1, step VIII). Afterward, remove the flask from the incubator and observe it under an inverted phase-contrast microscope to determine whether cells have attached (Fig. 1, step IX). If attached cells are observed, remove the old culture medium, wash with sterile 1 × PBS or Hanks' balanced salt solution (Solution 4), and add fresh DMEM working solution (Solution 1) with FBS (Solution 3) in the same proportion as before (Fig. 1, step X). Incubate the culture at 37ºC with 5% CO_2_. (P.S.: Comparable results can also be obtained using a non-CO₂ incubator, as cultures are maintained in DMEM supplemented with HEPES, which acts as a chemical buffer and maintains pH stability in the absence of CO₂ regulation. In this case, non-filtered culture flasks can be used).

#### Maintenance and subculturing of primary fibroblasts

Once the fibroblast culture is established, monitor daily for progression of cell confluency (i.e., an increase in cell density within the flask) and signs of contamination (Fig. 1, step IX). Replace the culture medium with DMEM working solution (Solution 1) every 2–3 days to eliminate metabolic waste and supply fresh nutrients (Fig. 1, step X). Upon reaching approximately 80% confluency (i.e., 80% of the surface area is covered with attached cells—Supplementary Material, Fig. S1), split the cells into two new subcultures (Fig. 1, step XI) following the same aseptic protocol used to initiate the culture. Place the following items inside the laminar flow hood: sterile Pasteur pipettes, cell culture flasks with vented polyethylene caps contain a 0.22 µm hydrophobic filter, and a waste bottle half-filled with 1–10% sodium hypochlorite. Remove the old cell culture medium and thoroughly wash the flask with 1 × PBS or Hanks’ balanced salt solution (Solution 4) for 30 s to remove all remaining FBS (Solution 3). Discard the solution and add 1 mL of trypsin–EDTA (Solution 6) to the cell culture flask. Orient the flask vertically so that the trypsin coats the entire growth surface and disrupts cell–matrix adhesions. Transfer the flask out of the laminar flow hood and gently tap its base to dislodge the remaining fibroblasts. Perform a check under an inverted phase-contrast microscope to confirm that all cells have detached. Return the flask to the laminar flow hood, add 2 mL of FBS (Solution 3) to deactivate the trypsin–EDTA, and then add 8 mL of DMEM working solution (Solution 1). Carefully resuspend the cell suspension by pipetting, then transfer 5 mL of this suspension into a new, clean cell culture flask. Place both flasks (original and subcultured) in an incubator at 37ºC with 5% CO_2_. Repeat this process until the desired number of cell culture flasks is obtained.

### Lymphocyte cultures

#### Sampling

Peripheral blood can be collected from either the jugular or the subalar vein, depending on the bird’s size, anatomy, and accessibility of the vessels. The choice of the most appropriate collection site should always be made by the veterinarian responsible for the procedure, in accordance with animal welfare and ethical guidelines. Before sampling, immobilize or anesthetize the animal in accordance with the approved ethical protocol. Perform antisepsis at the puncture site using 70% ethanol. Using a 1 mL syringe containing 30 µL of sodium heparin, collect at least 500 µL of peripheral blood; larger volumes are recommended to increase lymphocyte yield. Immediately transfer the blood into a heparinized tube and gently but continuously mix it for 1 min to prevent coagulation. If the sample must be transported for long distances, store it in a refrigerated container (4–10 °C) until laboratory processing.

#### Establishing lymphocyte cultures

**Fig. 2:**
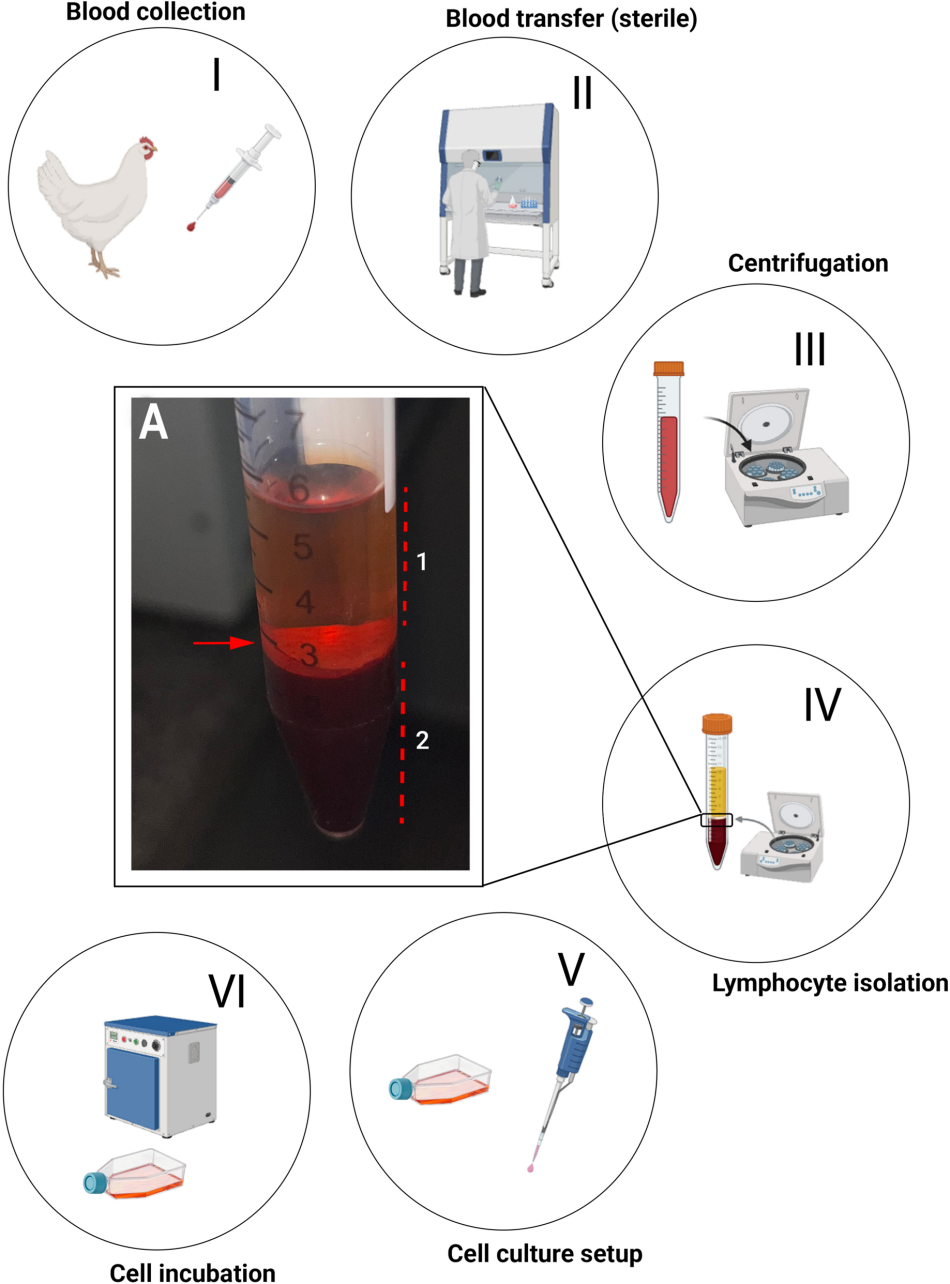
Workflow of lymphocyte cell culture. Schematic overview of the lymphocyte culture protocol from initial blood collection (I) to incubation period. II: Transfer the whole blood to a sterile 15 mL Falcon tube under a laminar flow cabinet. III: Centrifugation at 850 × g for 20 min (with the brake deactivated to preserve layer integrity). IV: Separation of the lymphocyte layer. V: Transfer of lymphocytes to supplemented culture medium. VI: Incubation of the culture at 40 °C for 42 h. A: Peripheral blood sample immediately after collection, following centrifugation. This process separates the blood into three distinct layers: the upper plasma layer (1), the intermediate buffy coat (enriched in leukocytes and platelets, indicated by the red arrow), and the lower layer of erythrocytes (red blood cells) (2). Detailed procedures for chromosome preparation are described in Fig. 4

To establish lymphocyte cultures, lymphocytes must first be separated from the erythrocytes and plasma present in whole blood (Fig. 2A). Before initiating the procedure, disinfect the laminar flow cabinet with 70% ethanol and place all required materials inside the sterile workspace, including sterile 15 mL centrifuge tubes, cell culture flasks, and micropipettes with sterile tips. Before starting the procedure, turn on the UV light for at least 25 min to ensure adequate sterilization of the working surface. Within the laminar flow cabinet, transfer the whole-blood sample into a sterile 15 mL centrifuge tube (Fig. 2, step II). Seal the tube and centrifuge at 850 × g for 20 min at room temperature with the brake turned off to preserve the integrity of the separated cell layers (Fig. 2, step III). Following centrifugation, return the tube to the laminar flow cabinet. Using a micropipette, carefully aspirate ~ 150 µL of the interphase layer containing the lymphocytes (Fig. 2, step IV). Transfer the collected lymphocytes to a cell culture flask containing pre-warmed supplemented culture medium (Solution 9) (Fig. 2, step V). Alternatively, when larger volumes of whole blood are available (> 4 mL), RPMI medium supplemented with PHA (Solution 10) can be used, yielding comparable results. Incubate the cultures at 40 °C for 42 h. To promote homogeneous cell growth and adequate nutrient distribution, gently mix the culture flask every 12 h throughout the incubation period (Fig. 2, step VI).

### Bone marrow cell culture

#### Sampling

**Fig. 3:**
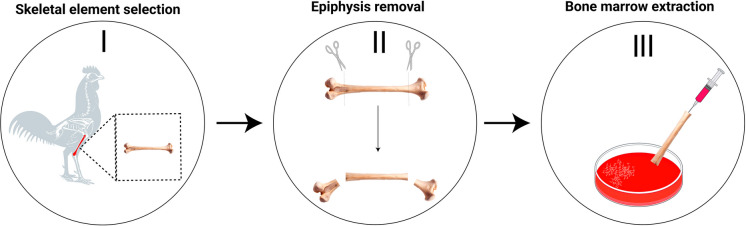
Workflow of the bone marrow isolation: Schematic overview of the bone marrow isolation procedure from the selected skeletal element. I: Bird skeleton with the tibiotarsus highlighted in red. II: Removal of the epiphysis from the tibiotarsus. III: Flushing of the bone marrow from the medullary cavity using a syringe into a Petri dish. Detailed procedures for chromosome preparation are described in Fig. 4

Suspensions of bone marrow cells are prepared from the tibiotarsus, the longest bone of the hindlimb. Animals must first be euthanized in accordance with the approved ethical protocol. The tibiotarsus, located distal to the femur, is dissected and excised, ensuring complete removal of surrounding muscle and skin tissues. Both epiphyses are then carefully removed to expose the medullary cavity containing the bone marrow cells required for obtaining mitotic chromosomes (Fig. 3). The bone cavity is flushed with ~ 8 mL of pre-warmed (37 °C) RPMI culture medium using a syringe, allowing the suspension to exit the opposite end of the bone into a glass beaker. To minimize dilution, the same medium should be repeatedly aspirated and reinjected until the bone marrow is completely removed (Fig. 3, step III). The resulting suspension is then homogenized by repeated aspiration and expulsion using a syringe.

## Preparation of mitotic chromosomes

**Fig. 4:**
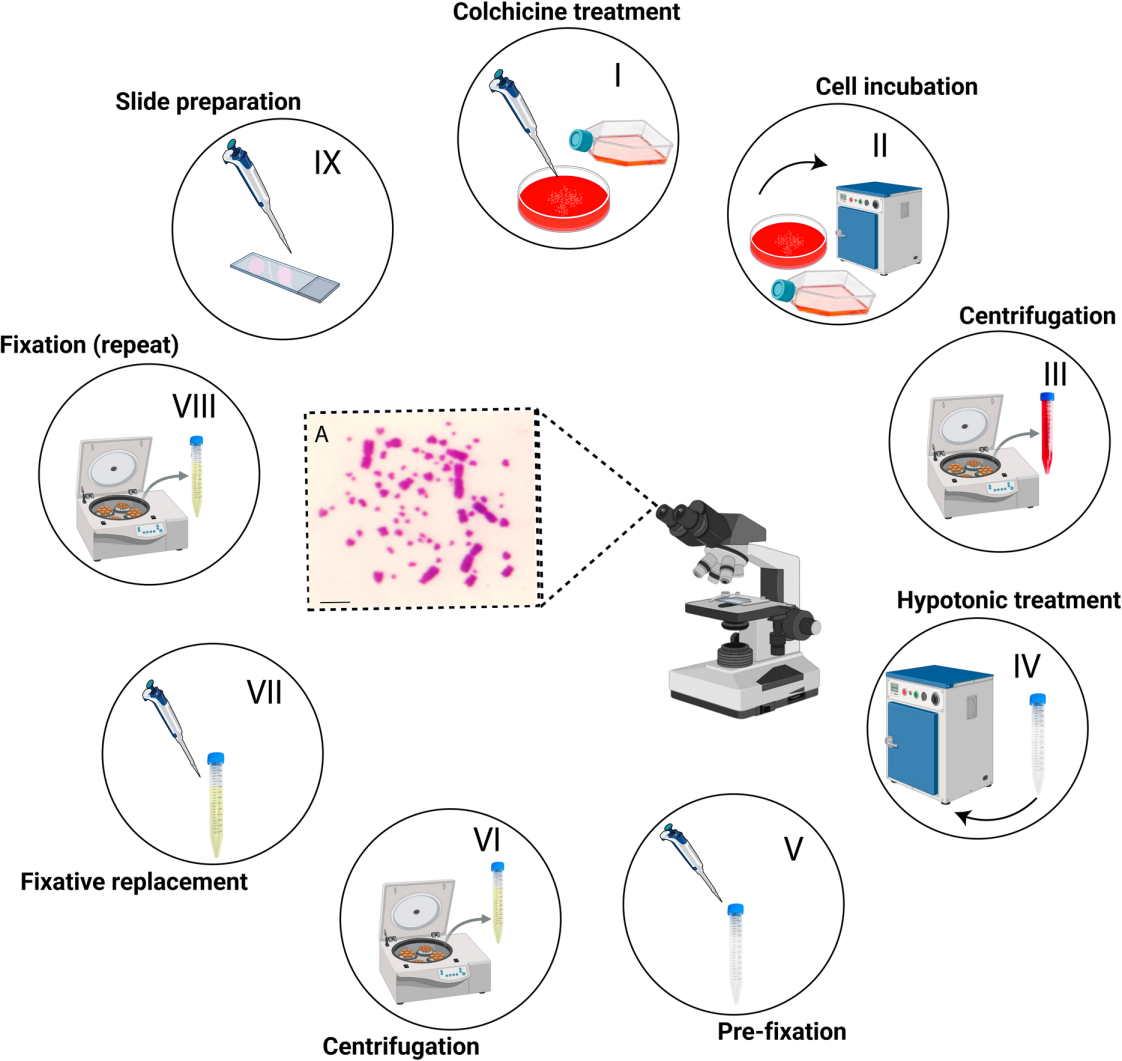
Workflow for chromosomal and slide preparation: I. Addition of colchicine solution to the medium culture or flask. II. Incubation of the cell suspension at 37 °C. III. Centrifugation of the cell suspension at 200 × g for 10 min. IV. Addition of hypotonic solution (Solution 12) followed by incubation at 37 °C for 30 min. V. Pre-fixation step: Add 1 mL of methanol-acetic acid (3:1) fixative solution (Solution 13). VI. Centrifugation of the cell suspension at 200 × g for 10 min. VII. Removal of the supernatant and addition of fresh methanol–acetic acid (3:1) fixative solution (Solution 13). VIII. Centrifugation, removal of the supernatant, and addition of 3 mL of methanol-acetic acid (3:1) fixative solution (Solution 13). IX. Dropping the cell suspension onto the microscope slide. A: mitotic metaphase chromosome spread (scale bar, 20 µm)

Mitotic chromosomes can be obtained from all three types of cultures described above, which serve as starting points for the chromosome preparation protocol. Regardless of the sampling source, chromosome harvesting follows the same standard. Briefly, cells are incubated with 0.01% colchicine to arrest cells in metaphase (Fig. 4, steps I-III), subjected to hypotonic treatment with the KCL solution (Solution 12) to promote chromosome spreading (Fig. 4, step IV), and subsequently fixed through repeated washes with cold methanol–acetic acid (3:1) fixative solution (Solution 13) (Fig. 4, steps V-VIII). Notably, colchicine can be replaced by colcemid (5.0 µg/ml), as both agents yield comparable results with similar incubation times (Kiazim et al. 2021).

For lymphocyte and fibroblast cultures, 100 µL of colchicine is added directly to the culture flasks. The cultures are then incubated for 1 h at 40 °C for lymphocytes and 37 °C for fibroblasts to allow metaphase arrest. For lymphocyte cultures, colchicine (Solution 11) should be added 1 h before the end of the 42—h incubation period. Following incubation, transfer the cells to a sterile 15 mL centrifuge tube. For bone marrow cultures, 80 µL of colchicine (Solution 11) is added directly to the bone marrow suspension in RPMI medium (Fig. 4, step I), followed by incubation at 37 °C for 1 h (Fig. 4, step II). During incubation, the suspension should be gently mixed every 15 min to ensure adequate dispersion and to prevent cell sedimentation.

Following the incubation with colchicine, transfer the suspension to a sterile 15 mL centrifuge tube and centrifuge at 100 × g for 10 min (Fig. 4, step III). In lymphocyte culture, after initial centrifugation, it is possible to see the buffy coat rich in leukocytes (for more information, see Supplementary Material, Fig. S2). For fibroblast cultures, cells must first be detached via trypsinization prior to transfer (see Maintenance and subculturing of primary fibroblast section). After detachment, collect the entire content of the culture flask and transfer it to a sterile 15 mL centrifuge tube (ensuring that no residual liquid remains in the flask—see Troubleshooting section, iii) and centrifuge at 100 × g for 10 min (Fig. 4, step III). Discard the supernatant (without disturbing the cell pellet) and add 10 mL of pre-warmed hypotonic solution (Solution 12), then incubate the samples at 37 °C for 30 min (for lymphocytes and bone marrow) and 8–10 min for fibroblasts (Fig. 4, step IV). Following hypotonic treatment, before centrifugation, add 1 mL of cold methanol–acetic acid (3:1) fixative (Solution 13) to each tube and gently mix by pipetting (Fig. 4, step V). Centrifuge at 200 × g for 8 min and discard the supernatant, leaving approximately 1 mL above the pellet (Fig. 4, step VI). Subsequently, add 10 mL of cold methanol–acetic acid (3:1) fixative (Solution 13), gently resuspend the pellet, and centrifuge at 200 × g for 8 min and discard the supernatant, leaving approximately 1 mL above the pellet. Repeat this washing step twice (Fig. 4, VII and VIII). After the final centrifugation and removal of the supernatant, add ~ 2 mL of methanol–acetic acid (3:1) fixative (Solution 13) (adjusted according to cell concentration), and resuspend the pellet thoroughly. The resulting cell suspension can be transferred to a sterile 1.5 mL microcentrifuge tube and stored at −20 °C or used immediately for slide preparation.

For chromosome preparations, 20 µL of the cell suspension is released using a Pasteur pipette onto different regions of a clean glass microscope slide preheated on a hot plate at ~ 50 °C (Fig. 4, step IX). Maintaining a temperature of 20–25 °C and relative humidity of 40–60% ensures consistent evaporation during droplet drying; deviations from these ranges may compromise spread quality. (PS.: The slide-dropping procedure can be performed within a fume hood; in our experience, as long as the airflow is moderate, it does not adversely affect chromosome spreading under standard working conditions). Preparations are then allowed to air dry and subsequently stained with Giemsa working solution (Solution 16) for 5–8 min. Finally, the slides are rinsed in running water and air-dried before analysis. (P.S.: Strict aseptic conditions are not required for most steps of mitotic chromosomal obtainment; however, trypsinization of fibroblast cultures should be performed in a clean laminar flow cabinet to maintain sterility).

**Fig. 5:**
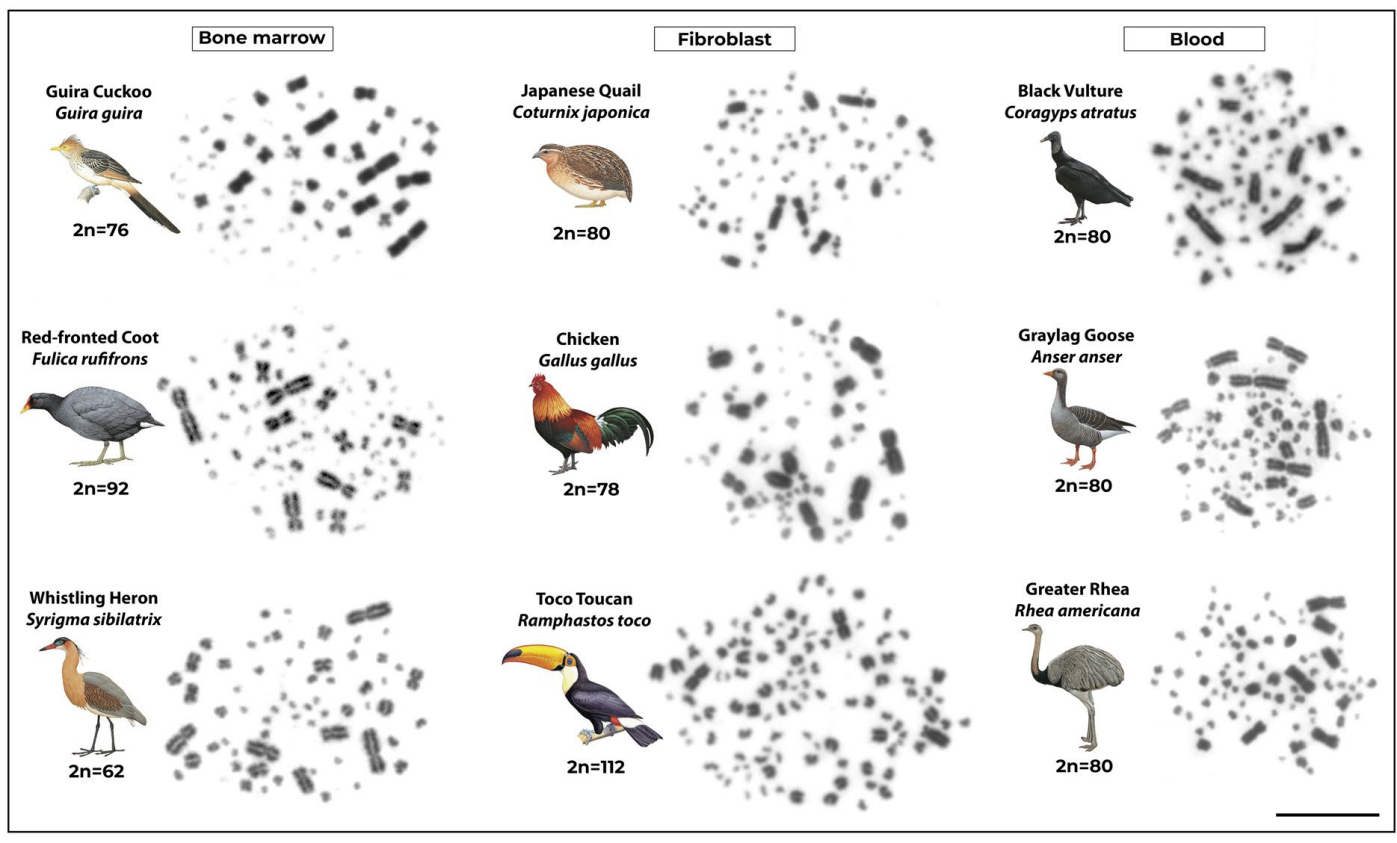
Metaphase plates of different bird species obtained from fibroblast, lymphocyte, and bone marrow cultures. Scale bar = 20 µm. Bird illustrations were obtained from Birds of the World (https://birdsoftheworld.org), accessed on 30 January 2026

Figure 5 highlights some results obtained using the abovementioned protocols in different bird lineages, in which the diploid number (2n) is indicated and was determined based on the analysis of at least 30 metaphase spreads per species.

## Troubleshooting

### Primary fibroblast culture

i) For optimal digestion of fibrous tissues (e.g., avian skin biopsies), the tissue should be finely minced and incubated with Collagenase Type I, an enzyme effective at degrading collagen-rich connective tissue. Digestion should be carried out for 3—8 h, with the duration adjusted according to the visual assessment of tissue dissociation. Less fibrous tissues typically require shorter digestion periods (3—4 h). If undigested tissue fragments or gelatinous/fibrous material remain after digestion, add DMEM medium supplemented with 1.5% antibiotics to a final volume of 10 mL in the Falcon tube. Homogenize the suspension, then transfer the supernatant to a new, sterile 15 mL Falcon tube, leaving the undigested pellet behind. The resulting cell suspension can then be processed following the standard cell culture protocol (Fig. 4).
ii) To minimize the risk of contamination, the laminar flow cabinet should be prepared before initiating step VII **(**Fig. 1). The work surface should be disinfected with 70% ethanol and all materials reorganized. Used reagents and any potentially contaminated items should be removed and properly discarded. The workspace should then be restocked with sterile cell culture flasks, Pasteur pipettes, and a discard container filled halfway with 1–10% sodium hypochlorite solution. Finally, the cabinet's UV light should be activated for at least 20 min before use to ensure adequate sterilization of the work area.
iii) If cells take longer than expected to reach the log phase (exponential growth), supplement the culture by adding 1–5% AmnioMAX II Complete Medium to the flask until visible proliferation is observed. This step is especially useful for skin biopsy–derived cultures, which typically exhibit slow growth during the initial weeks of cultivation.

### Lymphocyte cultures

iv) The volume of blood that can be collected varies among bird species and is constrained by their body mass. In general, a minimum body mass of approximately 100 g is required to safely obtain a 500 µL blood sample. This volume typically allows the preparation of only a single culture flask for lymphocyte cultures. In contrast, larger animals permit the collection of greater blood volumes (e.g., > 2 mL), yielding higher numbers of lymphocytes after centrifugation, enabling the establishment of multiple culture flasks and increasing the number of cytogenetic preparations obtained.
v) Comparable results can be obtained using either CO₂ or non-CO₂ incubators, as cultures are maintained in RPMI medium supplemented with HEPES, which acts as a chemical buffer and maintains pH stability independently of CO₂ regulation. In the absence of CO₂ incubation, culture flasks without filter caps can be used..

### Bone marrow

vi) Body size is a significant limitation for the success of lymphocyte and bone marrow cultures. In small birds, the reduced size of the tibiotarsus bone limits the total amount of marrow accessible for sampling. When the tibiotarsus is particularly large, however, each tibiotarsus should be processed individually in its own preparation to avoid excessive cell density in the culture medium. The resulting cell suspension can be diluted and distributed across multiple tubes during preparation.
vii) In some birds, excess adipose tissue may be present together with the bone marrow. In such cases, the preparation can proceed normally, and the adipose fraction should be removed after the first fixation step **(**Fig. 4 step V), which improves the quality of the resulting cell suspension.

## Discussion

Cell cultures are indispensable tools in modern cytogenetics, providing a reliable way to obtain high-quality mitotic metaphase spreads. Although a wide range of protocols have been developed for different animal groups over the years (Bertollo et al. 2015; Viana et al. 2016; Boroda et al. 2020; do Nascimento et al. 2024), including birds (Barcellos et al. 2022; Blank et al. 2025), comprehensive and accessible methodologies for obtaining avian chromosomes remain surprisingly scattered. This scarcity of integrated protocols represents a steep barrier for laboratories and researchers new to the field, contributing to inconsistencies in data quality and reproducibility. The protocols presented here address this gap by detailing a robust step-by-step optimized workflow for the three main methodologies employed in birds' cytogenetics, thereby ensuring reliable, high-quality chromosomes suitable for any downstream applications. The complexity of avian karyotypes, characterized by the presence of numerous microchromosomes, poses significant challenges for accurate identification (Griffin and Burt 2014). High-quality chromosomal preparations are therefore essential not only for determining diploid numbers and reliably distinguishing macro- from microchromosomes, but also for enabling advanced cytogenetic analyses, such as the detection of structural rearrangements and the characterization of karyotype organization (Völker et al. 2010). In the context of cytogenomics, well-resolved metaphase spreads provide a critical framework for validating genome assemblies. Accordingly, the development and dissemination of robust, high-resolution cytogenetic protocols are key to strengthening the integration of cytogenetic and genomic approaches in avian research.

Obtaining chromosomes through peripheral blood lymphocyte culture is a practical and effective approach in avian cytogenetics, especially for endangered species. Blood collection is relatively simple and minimally invasive, not requiring strict aseptic procedures beyond the usual precautions to avoid contamination. Belterman and De Boer (1984) tested Concanavalin A (ConA) and Lipopolysaccharide (LPS) separately and concluded that they were not effective for lymphocyte culture in birds. However, our results indicate that the combination of ConA and LPS as mitogenic stimulants indeed results in good-quality chromosomal preparations across different avian groups, ranging from ratites to Passeriformes. The combination of ConA and LPS targets both lymphocyte populations, as ConA primarily activates T lymphocytes, whereas LPS preferentially stimulates B lymphocytes (Andersson and Melchers 1974). Consequently, this combination increases the number of cells entering division, leading to a higher mitotic index and an increased yield of metaphase cells.

Lymphocyte cultures also provide practical advantages over alternative methodologies. They are comparatively less labor-intensive and require fewer reagents, as the culture medium typically does not need to be replaced during the procedure. This reduced level of manipulation largely confines most contamination risks to the sample collection stage, minimizing opportunities for secondary contamination. However, the success of peripheral blood cultures ultimately depends on the presence of circulating lymphocytes that retain the capacity to proliferate under in vitro conditions. In birds, lymphocytes are the main leukocyte type with this capacity (Davis et al. 2008). Consequently, natural variation in lymphocyte abundance among individuals can substantially influence the success and yield of peripheral blood cultures. Samples with fewer lymphocytes yield a diminished reservoir of cells responsive to mitogenic stimulation, potentially leading to a decreased count of observable metaphases. Moreover, avian leukocyte profiles are highly variable and may shift with species, age, physiological conditions, and environmental stress (Davis et al. 2008). Furthermore, capture and handling stress can induce hematological changes, including increases in heterophil counts and concomitant reductions in lymphocyte abundance (Gross and Siegel 1983). Such shifts may influence culture performance and likely contribute to the variation in metaphase yield observed among samples.

In contrast, fibroblast cultures are usually more resource-intensive and laborious, requiring regular monitoring, medium changes, and subculturing. However, they provide a cleaner chromosome suspension free from contaminating cell types, as well as far greater flexibility for future analyses (Barcellos et al. 2022; Blank et al. 2025). These include the creation of renewable biobanks, the cryopreservation of genetic material from deceased or rare specimens, and the capacity to conduct functional assays that are not feasible with short-lived lymphocyte cultures or bone marrow cell suspensions (Blank et al. 2025). These advantages make fibroblast cultures especially valuable for future cytogenomic investigations. Since the refinement of genome assembly pipelines, chromosome-level assemblies are increasingly being published (Wellcome Sanger Institute 2026). Cytogenetic data provides an essential complementary framework for interpreting these assemblies by enabling independent verification of diploid chromosome numbers and large-scale chromosomal organization. The ability to establish and maintain a long-lasting cell line of a particular species of interest may prove critical not only for validating genome assemblies but also for providing resources for studying gene expression and cellular function. However, prolonged culturing and repeated passaging can lead to the accumulation of chromosomal abnormalities and genomic instability, which in mammalian cells may arise after relatively few passages (e.g., ~ 6–10) (Puck et al. 1958; Sax and Passano 1961). Regular monitoring of karyotypic stability is therefore essential to avoid potential biases in subsequent analyses.

Another common source of mitotic chromosomes in avian cytogenetics is bone marrow preparations. Hematopoietic tissues contain groups of cells that are actively dividing, so this method has the advantage of providing a naturally high number of dividing cells (Sharma & Sharma 1980; Rooney & Czepulkowski 1992). As a result, bone marrow procedures frequently produce a large number of metaphase spreads in a very short processing time without requiring in vitro mitogenic stimulation. This fact makes the technique particularly useful for rapid cytogenetic screening or immediate chromosomal preparation (Ray-Chaudhuri 1976). However, this process is intrinsically more invasive and typically necessitates the sacrifice of the specimen or the collection of new material from recently deceased individuals. Furthermore, bone marrow preparations often contain a mixture of hematopoietic cell types (Rooney & Czepulkowski 1992; Kaushansky et al. 2016), which can increase background material and occasionally hinder the identification of well-spread metaphases. Consequently, although bone marrow protocols are efficient and relatively straightforward, their application may be limited to studies that do not involve rare, protected, or otherwise valuable specimens, where non-lethal sampling approaches are needed.

## Conclusions

In summary, fibroblast cultures generally produce the highest-quality metaphase spreads, characterized by well-dispersed and extensively elongated chromosomes that substantially improve cytogenetic resolution and karyotyping accuracy. These characteristics render fibroblast-derived preparations especially appropriate for comprehensive chromosome analyses and detailed cytogenomic applications. However, this approach requires more extensive laboratory infrastructure, longer culture periods, and greater reagent consumption than other methods. In contrast, bone marrow and peripheral blood lymphocyte preparations are faster and less resource-intensive, enabling the rapid generation of metaphases with relatively simple laboratory procedures. These characteristics make them well-suited for routine cytogenetic screening, large-scale surveys, and studies conducted under limited logistical conditions. Ultimately, the choice of methodology depends on the specific objectives of the study, the availability of biological material, and practical considerations related to time, infrastructure, and ethical constraints associated with sample collection.

## Supplementary Information

Below is the link to the electronic supplementary material.

Supplementary file1 (PDF 10555 KB)

Supplementary file2 (PDF 4981 KB)

## Data Availability

All data supporting the findings of this study are included in this published article and its supplementary information files.

## Abbreviations

2n: Diploid number
°C: Degrees Celsius
CO₂: Carbon dioxide
ConA: Concanavalin A
DNA: Deoxyribonucleic acid
DMEM: Dulbecco’s Modified Eagle Medium
EDTA: Ethylenediaminetetraacetic acid
FBS: Fetal Bovine Serum
G: Relative centrifugal force (gravity)
HEPES: 4-(2-Hydroxyethyl)-1-piperazineethanesulfonic
KCl: Potassium chloride
LPS: Lipopolysaccharide
mL: Milliliter
Mb: Megabase
µL: Microliter
µm: Micrometer
PBS: Phosphate-Buffered Saline
PHA: Phytohemagglutinin
RPMI: Roswell Park Memorial Institute medium
ZW: Sex chromosome system
