# Supplementary figures and images for: Bridging methodological gaps in avian cytogenetics: comprehensive and optimized protocols for chromosomal preparation in birds
Source: Chromosome Res. 2026 May 18;34(1):11. doi: 10.1007/s10577-026-09803-8 (PMC13180766; doi:10.1007/s10577-026-09803-8)

**A**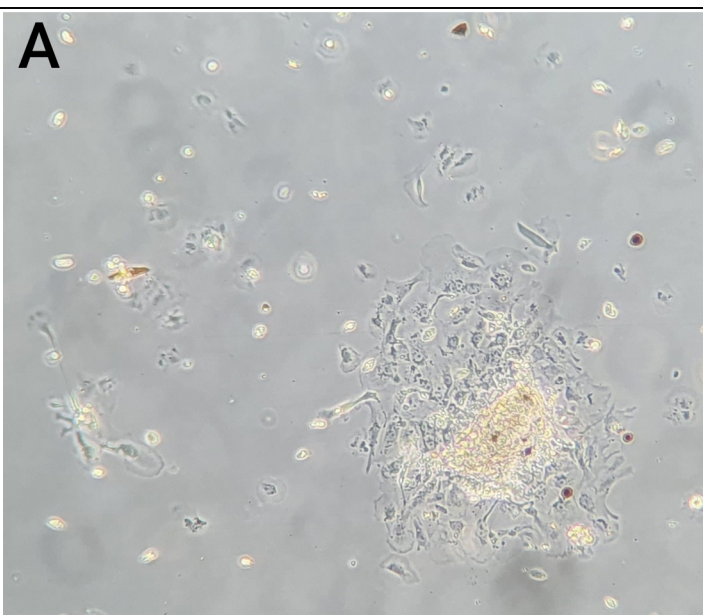**B**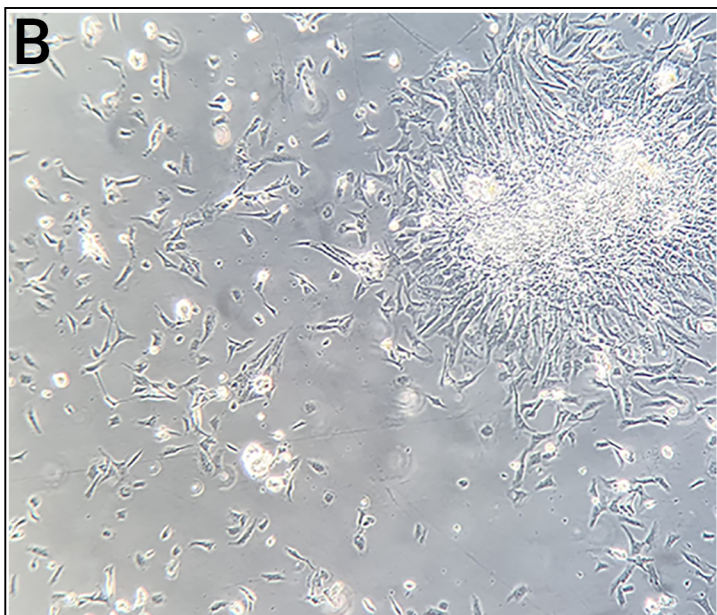**C**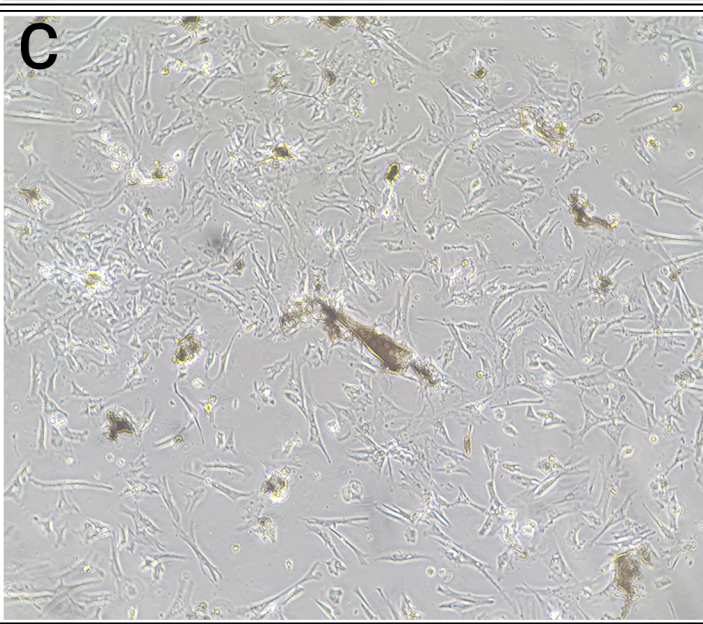**D**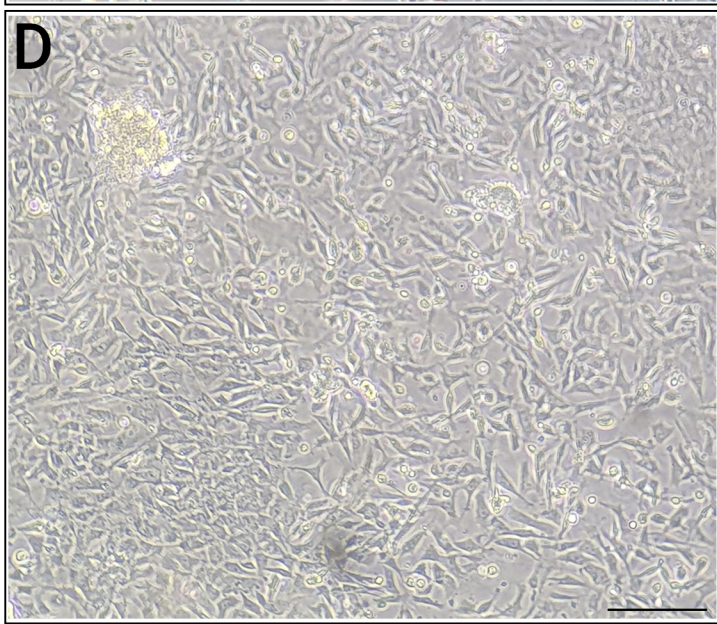

Supplement: Supplementary file 1 — Supplementary file1 (PDF 10555 KB) [file 10577_2026_9803_MOESM1_ESM.pdf]

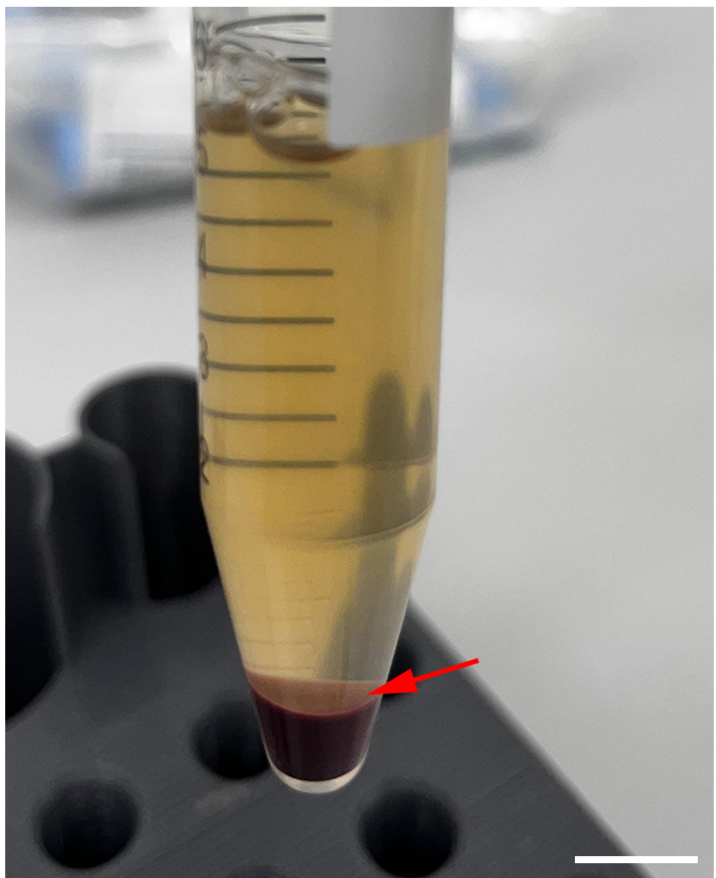

Supplement: Supplementary file 2 — Supplementary file2 (PDF 4981 KB) [file 10577_2026_9803_MOESM2_ESM.pdf]
